# Communication About Chronic Pain in Older Persons' Social Networks: Study Protocol of a Qualitative Approach

**DOI:** 10.3389/fpubh.2021.764584

**Published:** 2021-11-03

**Authors:** Gilles Merminod, Orest Weber, Carla Vaucher, Imane Semlali, Anamaria Terrier, Isabelle Decosterd, Eve Rubli Truchard, Pascal Singy

**Affiliations:** ^1^Liaison Psychiatry Service, Department of Psychiatry, Lausanne University Hospital, Lausanne, Switzerland; ^2^Institute of Social Sciences, Faculty of Social and Political Sciences, University of Lausanne, Lausanne, Switzerland; ^3^Pain Center, Service of Anesthesiology, Lausanne University Hospital and University of Lausanne, Lausanne, Switzerland; ^4^Geriatrics and Geriatric Rehabilitation Service and Chair of Geriatric Palliative Care, Department of Medicine, Lausanne University Hospital, Lausanne, Switzerland

**Keywords:** communication, chronic pain, geriatric medicine, social network, qualitative research

## Abstract

A lack of social relations appears to impact on health and life expectancy among the older persons. The quality and diversity of social relations are correlated with good health and well-being in later life. Chronic pain is a crucial issue in aging population. Effective communication between the older persons with chronic pain, their relatives and the actors of the healthcare system facilitates the management of this condition. Studies on communication in later life generally do not consider the older persons' social network as a whole, focusing only a specific segment (e.g., family or medical staff). This lack of scientific data prevents the actors of the healthcare system from offering solutions to bridge clinically relevant communication gaps. As a consequence, our study has three objectives: (1) to identify how the older persons perceive communication about chronic pain with their social network; (2) to identify their unmet communication needs; (3) to develop recommendations that improve communication about chronic pain in later life. The study will be divided into two phases. The first phase will meet objectives 1 and 2. It will involve individual interviews with about 50 people over 75 years old suffering from chronic pain and without major cognitive or auditory troubles. In this phase, we will apply a multi-layered analysis. We will map the older persons' personal network and identify their communication practices and needs, by combining content and discourse analysis with social network theories. The second phase of the study will aim at recommendations based on the results of the first phase (objective 3). It will require focus groups with different sets of stakeholders (older persons, relative caregivers, health professionals, decision-makers). In the second phase, we will use content analysis to pinpoint the concerns and suggestions for action. The results will be disseminated on three levels: (1) to the scientific world (specialists in the field of health and aging and health communication); (2) to health practitioners working with older persons; (3) to society at large, with a focus on institutions and groups directly concerned by the issue.

## Introduction

Chronic pain affects a large proportion of people over 65 years old, with figures ranging from just under a third to over four-fifths of this group (1). Chronic pain is a major factor of vulnerability among the older persons (2). When poorly managed, it is associated with excessive inactivity leading to physical deconditioning, increased mortality, a decreasing quality of life and several complications (3). The latter includes functional decline, that is to say the development of impairments in the ability to perform basic activities of daily life (4), impairments of cognitive and psychomotor functions, sleep and appetite disorders, and an increased risk of anxiety and falls (5–8). The association of chronic pain with a multiplicity of disabilities (reduced mobility, sleep disorders, depression, etc.) makes it difficult to manage on a daily basis. It affects both the persons suffering from chronic pain, the relationships they have with the members of their social networks, and these members themselves (9).

Fluid communication between the older persons suffering from chronic pain, their relatives and the actors of the healthcare system facilitates the management of this condition in later life (2, 10) and leads to better outcomes in terms of pain self-management, general health and quality of life (11, 12). Through communication, the older persons can get the help and care they need, and in particular access the relevant information as well as the techniques and treatments of pain reduction. Without appropriate or sufficient communication, they run the risk of over- or under-using health services, of being less likely to adhere to treatment and of being less involved in the management of pain (13). As a consequence, older persons suffering from chronic pain need to have the resources to communicate about their health problems with the members of their social networks (family, friends, health professionals, etc.), whether it be to find medical and psycho-social information or social support.

Social relations are key for the promotion of health and the prevention of disease (14, 15), and, unsurprisingly, relations with family and friends impact on the general health status of the older persons (16). For instance, the integration of older persons in a family structure tends to have a favorable impact on their objective or perceived health status (17–21). A high degree of trust in others and multiple opportunities for social participation are often correlated with a better assessment of one's own health status (22) and a better sense of well-being (23, 24). On the contrary, a lack of social relations appears to accelerate functional decline and to weigh on depressive symptoms and mortality among the older persons (25, 26). More than their quantity, the quality and diversity of social relations seem to be factors correlated with good health and well-being in later life (27, 28).

These studies generally do not include health professionals as a fully-fledged part of the relational ecology of the older persons, thus introducing a problematic cleavage within the older persons' social networks. Moreover, they do not focus specifically on communication within the social network of older persons suffering from chronic pain, whereas it seems valuable to better understand who becomes -and who does not become- a resource relating to health in the context of interpersonal interactions, how and why. This significant lack of scientific data on communication about chronic pain in later life prevents the actors of the healthcare system from being able to offer solutions in line with the realities experienced and perceived by older persons suffering from this condition. As a consequence, our study, at the intersection of human and medical sciences, has three objectives:

(1) to identify how the older persons perceive communication about chronic pain with the members of their social network, with a particular focus on communication problems and their medical and social implications;
(2) to identify the unmet communication needs of the older persons suffering from chronic pain;
(3) to develop lines of action to support communication about chronic pain in the older persons' social networks in the French-speaking part of Switzerland, with the help of various groups of stakeholders (health care professionals, older persons suffering from chronic pain, policymakers, etc.).

## Methods and Analysis

### Setting

This study will be carried out by a multidisciplinary team of experienced researchers (medical staff as well as linguists and psychologists) within the Psychiatry Liaison Service of the Lausanne University Hospital. The project will be steered by an advisory board gathering medical and nursing staff, experienced researchers, and decision-makers working in the field of health in later life. The team will collaborate with several institutional bodies in the French-speaking part of Switzerland, all focused on the management of health in later life.

### Patients and Public Involvement

The development of the research questions and of the study design has been informed by a pilot study with nine persons over 75 years old suffering from chronic pain in 2016–2017. In this regard, it draws on current frameworks in participative action research in public health (29). Following a grounded theory perspective (30, 31), the research questions and the study design have been adapted step by step to people's priorities, experiences and preferences. The research team has greatly benefited from discussions with these persons and is grateful for their contributions. The public will be involved in the recruitment of new respondents through the so-called snowball technique (participants already included in the study will indicate potential new respondents). In addition, the study design itself will foster public involvement inasmuch as its first phase (semi-structured interviews with older persons suffering from chronic pain) will lead to a second phase (focus groups with different stakeholders, including older persons with chronic pain) aiming at recommendations in line with people's experiences and needs. In the very course of the study, the focus groups will be a first site for the dissemination of the results of phase 1, by making participants aware of some aspects of the issue. At the end of the study, the overall results will be disseminated to study participants through lay publications and public events (see section Dissemination).

### Study Design

The study design was submitted, assessed and approved by the Swiss National Science Foundation (Grant: no. 10001C_179292), through an external and internal peer-reviewing process that checked the originality, scientific relevance, quality and topicality as well as the methods and the feasibility of the project.

#### Theoretical Foundations

The study design rests on the fact, evidenced long ago in language sciences (32, 33), that communication is not just an exchange of information, but meets practical and relational ends within specific social situations. From this vantage point, the disclosure of chronic pain in a talk, for instance, may follow a situated cost/benefit evaluation (34). A social situation necessarily entails explicit and implicit norms. To a large extent, these govern what can be communicated, to and by whom, how and what for (35–37). Norms vary according to the profile of those communicating (life history, profession, knowledge and experience, more or less valued or stigmatized social memberships). In spite of being of the same generation, older persons are not a socially homogenous group. They have multiple memberships and identities (38, 39), which may influence the way they communicate about chronic pain.

Communication necessarily takes place in a set of relations between individuals, namely a social network (40). Depending on the person, their social network varies in size and density but also in terms of the explicit and implicit norms governing verbal communication (41). In addition to being more or less appropriate spaces to talk about chronic pain, social networks can also be more or less useful to individuals in the management of their chronic pain. In other words, they are part of the individuals' social capital (35). In this regard, the multiplexity of social networks—that is, the potential combination of several social roles within the same relationship (for instance, individual A and individual B are bound by a doctor/patient relationship but also by a family or a neighboring tie)—might well contribute significantly to the efficiency of a social network in the management of chronic pain (42).

#### Overall Research Design

A qualitative approach will enable a detailed analysis of the issues relating to communication about chronic pain in later life (10, 43). It will allow the uncovering and systematization of explicit and implicit communicative norms. The data will be produced through semi-structured interviews. Such data will give access to the individuals' knowledge and perceptions about one or several aspects of their existence (44). The study will be divided into two phases.

The first phase (phase 1), with a duration of 18 months, will meet the first two objectives of the study. It will answer the following research questions: Is chronic pain a topic within older persons' social networks? If so, when, how and why? What are older persons' specific communicative needs when it comes to talk about their chronic pain? To do so, phase 1 will involve carrying out individual interviews with about 50 people over 75 years old.

The second phase of the study (phase 2) will aim at formulating recommendations based on the results of phase 1 and supporting communication about chronic pain in older persons' social networks (objective 3). Its duration will be of 18 months. It will be focused on the following research questions: What are the health care system's answers to the detected gaps and needs? What new institutional and educational means could we suggest? To answer these questions and meet objective 3, phase 2 will require to carry out collective interviews, namely focus groups, with different sets of stakeholders (older persons, relative caregivers, health professionals, decision-makers).

At the end of the study, the dissemination of the results and recommendations will aim at generating positive changes in practice and policy: both by raising awareness of specific concerns associated with chronic pain in later life and by highlighting practical solutions from the field that can be developed on a larger scale.

### Data Collection

#### Data Collection in Phase 1: Individual Interviews

In phase 1, the research team will carry out semi-structured interviews with about 50 individuals over 75 years old. The interviews will bring together one researcher with one older person in face-to-face interactions (one-on-one interviews), and will be audio-recorded. Given the scope of investigation, all the interviews will be divided into two parts (part 1 and part 2), separated by a few days. In addition to the usual questioning strategies used in semi-structured interviews on health topics (45, 46), the research team will use a concentric circle methods, inspired by the hierarchical mapping technique (47, 48), which allows for a systematic investigation and description of the different ties individuals have in their social network. This method has been successfully applied to a group of older persons in a previous study (48) as well as in our pilot study. The interview guide may be refined during the course of the study in an iterative spiral process (30), which is why we will proceed by successive interview campaigns (from 10 to 10) alternating with intermediate analyses. This segmentation into two parts will also contribute to strengthening the mutual trust between the interviewee and the interviewer, which adds depth to the data, as observed in our pilot study.

To reduce the risks of social desirability bias and of the impact of the researchers' preconceptions (49, 50), the interview guide is conceived so that the interviewees will not perceive the answers potentially favored by the interviewers. Researchers will gather information about possible bias through a systematic reflexive feedback on their practice, especially through detailed notes after each interview, which will feed future discussions of the results and limitations of the project. The interviews will be carried out at the interviewee's home or in quiet premises in their care homes, with the aim that they feel free to comment on the health care system and institutions. Hospital or association premises might also be used if the interviewee does not wish to host the researcher.

Part 1 of the interview (45-60 min) will firstly aim at getting to know the interviewees, by collecting social and biographical data through their life story (self-presentation, what has mattered in their life and what matters today). This first step will help understanding their communicative ecology (51, 52), particularly what the interviewees' priorities are in relation to the research topic. Part 1 will also focus on the interviewees' chronic pain (description of its localization, intensity, duration, management, etc.) and its consequences on their everyday life at functional, relational, emotional and cognitive levels (e.g., inability to go shopping, frustration of constant suffering, etc.). At the end of part 1, the interviewees will draw a concentric map of their social network: ego (the interviewee) being in the center of the map surrounded by the alters (the members of the interviewee's social network) arranged in order of importance (important+, important, important-, unimportant). The interview will be still recorded while the interviewees are drawing their map, giving them the opportunity to comment on the ties between them and the members of the network, or between the members of the network themselves. With such a method, social networks will be only apprehended from the perspective of a focal actor (ego). It will allow the identification of the ties between egos and the members of their entourage as well as their perceptions of these ties (53–55). This will be particularly relevant to this project, which aims to understand what makes the older persons communicate about chronic pain with those around them or not and to what extent these persons become a resource for the older persons.

Part 2 of the interview will be carried out a few days after part 1 and will last around 1 h. It will aim at addressing in detail the interviewees' communication about their chronic pain with the members of their social network. Communication with each member of the interviewee's social network will be investigated: frequency of interactions about chronic pain if existing, communicative agenda of the persons taking part in the interactions, communicative difficulties and preferences, facilitators and barriers, as well as costs and benefits of communicating about their chronic pain, communicative strategies to talk about their chronic pain or to avoid the topic. At the end of part 2, the interviewees will be asked about their communicative needs and expectations relating to chronic pain.

#### Sample in Phase 1: Eligibility Criteria and Recruitment

The sample will consist of around 50 persons. However, after 40 persons, the research team will stop recruiting new participants if a thematic saturation effect on the entire sample were to be reached (56). The participants should be aged 75 and older, which is a significant stage in terms of functional limitations and prevalence of chronic pain (4, 57). The participants should live in the French-speaking part of Switzerland to ensure a form of cultural and linguistic comparability. They should suffer from chronic pain, generally defined as pain that lasts more than 3 months (58, 59). Because of the method of investigation (interviews), the participants should not suffer from major cognitive or auditory impairments.

The relatively large number of participants is not motivated by a probabilistic logic but by the need to ensure sufficiently diverse profiles within the sample. This is the condition to be able to observe potential trends related to social affiliations known to affect the health care and communication, such as gender, ethnicity, socioeconomic level, and age (60). The variables of place of residence (living at home or in a care home), the fact of living alone or with somebody, and having children or not will be added to the study criteria, based on previous studies (10, 43, 61–63) and on the observations made during the pilot study. The sample should encompass at least 10 individuals belonging to the same group for each criterion (for instance, at least 10 men out of the 40 participants recruited, at least 10 persons whose primary socialization was not in Switzerland, at least 10 persons without children, etc.). This threshold of 10 persons should allow to obtain a critical mass of discourse per group that is sufficient to identify possible tendencies toward consensus or dissension within each group.

The recruitment will rely on the medical staff being part of the research team as well as on the members of the advisory board who work with older persons. Part of the interviewees will be recruited in geriatrics and nursing homes as well as in a pain clinic. Another part will be reached through associations, churches and home care. The rest of the sample will be constituted using the so-called snowball technique, that is to say, the indication of new respondents by participants already included in the study. This process will enable to reach subjects who are potentially less in contact—or even in difficulty—with the health care system. By doing so, the study will try to avoid the main methodological bias of studies that entrust the selection of participants mainly to clinicians, who often tend to choose people with whom they have good relationships (64). This combination of recruitment strategies worked well in the pilot study.

#### Data Collection in Phase 2: Focus Groups

In phase 2, the research team will carry out several focus groups with sets of stakeholders (older persons, relative caregivers, health professionals, decision-makers). Focus groups allow the observation of interactions between participants, the formation and negotiation of opinions in real time, and the sharing of experience between people that have common interests or experience similar life situations (65, 66). This method appears particularly relevant for developing recommendations that are in line with the existing dynamics in the field under study. During the focus groups, a selected set of results produced in phase 1 will be presented to the participants and compared with key elements of the pre-existing literature on chronic pain communication. The focus groups will aim at identifying the needs and possibilities of interventions in clinical, associative, public and personal settings. These might be oriented toward adapting professional practices or modifying perceptions, whether of public opinion or of specific segments of the population.

In accordance with recommendations found in literature, each focus group will include between 6 and 10 participants and will last between 60 and 90 min (67–69). Likewise, the audiences gathered in the groups will be as homogeneous as possible from the point of view of their relation to the topic and, where appropriate, their professional profiles, in order to encourage as much freedom of expression as possible (e.g., to avoid interviewees having to criticize the medical world in front of doctors). The focus groups will be audio-recorded. They will be led by two members of the research team, one of them leading the discussion, the other taking additional notes in order to facilitate the transcription of the discussions and guarantee the completeness of the questioning. Meetings will be held in venues that encourage participation (e.g., hospitals for clinicians only).

#### Sample in Phase 2: Eligibility Criteria and Recruitment

The composition of the focus groups will be based on the pre-existing literature on chronic pain communication (43, 70–74) and on the pilot project that show the importance of the 8 following groups on issues related to chronic pain in later life: (1) older persons who suffers from chronic pain, (2) (family) caregivers, (3–6) doctors and other healthcare professionals working in hospital and ambulatory care, (7) members from community-based associations, and (8) policy-makers.

These 8 groups have been defined in order to obtain the views of parties that have different relationships to chronic pain in later life and its management, either because they suffer from chronic pain themselves or because they have a relative or close friend who suffers from it, or because they work on a day-to-day basis with older persons suffering from chronic pain, or because they can influence perceptions and practices at a decision-making level. The involvement of medical staff in the research team and of decision-makers in the advisory board will be an insider anchoring that will ensure the feasibility of recruitment.

### Analysis

#### Analysis in Phase 1: Mixing Discourse and Content Analysis With Social Network Theories

In phase 1, the research project will apply a multi-layered analysis mixing discourse and content analysis with social network theories. It will aim at mapping the older persons' personal network and identifying their communication practices and needs, combining content and discourse analysis with social network theories. The analysis of the interviews transcriptions will be carried out through the qualitative data analysis software NVivo. The research team will fill a catalog of methodological notes during the analyses, with a view to future discussions of the results and limitations of the project.

Content analysis (75, 76) will inventory and describe the semantic categories available among the participants relating to communication about chronic pain. Following an inductive reasoning and an iterative spiral process (30), the coding strategy will rely on a process of intercoder agreement repeated all along phase 1. The categories used for coding will emerge from the data, and will only then be compared with preexisting theories. The distribution of semantic categories will then be considered in comparison with the interviewees' social memberships (gender, age, etc.).

Discourse analysis (77, 78) will support content analysis to identify explicit and implicit norms relating to communication about chronic pain in later life. Discourse analysis will allow the researchers to apprehend the rhetorical logic and the sequential organization of the interviewees' talk through the study of the linguistic forms and patterns they used. By doing so, the analysis will go beyond the sole literal meaning of words, which is usually pointed as the main limitation of content analysis (79, 80). Discourse analysis will also prove useful to identify how the interviewees manage language resources to situate themselves in relation to collective identities (81), as in the case of claiming to be part the “young-old” persons in contrast of the “old-old” ones.

Social networks theories (82), and particularly the analysis of personal networks visualizations (83), will allow the research team to describe how the interviewees perceive their social network. Such an analysis will offer a description of social networks structure in terms of type of ties (close and extended family, friends, neighbors, etc.), size (number of members in the network) and density (here, based on the difference between members considered as important vs. those seen as unimportant). This description, enriched by the results of content and discourse analysis, will lead to a heuristic typology of social networks relating to communication about chronic pain in later life.

In a nutshell, the expected outcomes of the analysis in phase 1 are: (1) the mapping of personal networks as they are perceived by the interviewees; (2) the description of the place given to communication about chronic pain in these networks; (3) the identification of the barriers and facilitators to communication about chronic pain; (4) the identification of communication needs relating to chronic pain.

#### Analysis in Phase 2: Content Analysis Leading to Practical Recommendations

The focus groups transcripts will be subject to content analysis, as described in phase 1. The analysis will focus on the concerns and suggestions for action brought out by the participants, considering the convergences and divergences emerging within as well as between the focus groups (66). The research team will favor an intergroup perspective that flags the concerns and suggestions shared by different groups of stakeholders in the management of chronic pain in later life (see section Sample in Phase 2: Eligibility Criteria and Recruitment). The later, because they are shared, could prove particularly interesting in terms of feasibility.

The expected outcome of the analysis is the development of recommendations that promote practices suited to the communicative and relational needs of older persons suffering from chronic pain. The focus groups will facilitate the formulation of recommendations that will not only be in line with the results of phase 1 (which will relay the voice of the older persons suffering from chronic pain) but also with the concerns of the other stakeholders on this topic.

## Discussion

This article details the protocol of a qualitative study that investigates the communication about chronic pain in older persons' social networks. Its design in two phases will produce two types of results that will be tightly interlaced: phase 1 will allow us to better understand what it is like to communicate about chronic pain in later life; phase 2 will allow us to develop recommendations that integrate the older persons' needs and the concerns of other stakeholders (relatives, health practitioners, etc.). By doing so, the study will take the very social ecology of chronic pain communication into account rather than considering the expression of chronic pain as a mainly individual problem. In addition, the study will focus on the communication within the older persons' overall social network, without favoring one kind of social ties (e.g., family) over another (e.g., healthcare professional).

Carried out by a multidisciplinary team of researchers in collaboration with several institutional bodies involved in the management of health in later life, this study is largely inspired by action research (84–86), which aims to produce knowledge with the active participation of stakeholders (87, 88) while seeking to modify some of their perceptions and practices (31).

## Dissemination

The results will be disseminated on three levels: (1) to the scientific world, particularly to specialists in the field of health and aging, health communication, language sciences and social networks theories; (2) to health practitioners working with older persons; (3) to society at large, with a focus on institutions and groups directly concerned by the issues relating to chronic pain and health in later life.

(1) The results will be published through four scientific articles in international and national peer-reviewed journals as well as in at least six scientific conferences in the field of medicine, language and communication, and social networks research. The research team will also organize a scientific event on the topic at the end of the project.
(2) The results will be communicated in health professional journals and on the occasion of professional trainings in Switzerland. They will also be disseminated in the form of recommendations addressed to the authorities concerned. This will be facilitated by the involvement of medical and nursing staff in the project as well as that of decision-makers in the advisory board. The participation of health practitioners from different disciplines in the focus groups will also enable a disseminating effect at the local level, including through informal talk with colleagues who are not part of the research project.
(3) The results will be disseminated through lay publications and media appearances, with an institutional and associative support. A press conference and an event for the general public will be organized to raise public awareness about the living conditions of the older persons suffering from chronic pain. If the results show a pressing need to change some of the norms governing communication about chronic pain in later life, the research team will implement a new project, chiefly focused on the modification of perceptions and practices relating to communication about chronic pain in the general public.

## Ethics Statement

The study design has been approved by the Cantonal Commission on Ethics in Human Research (CER-VD: 450/15; 15.11.2018). All the participants will be required to give their written consent after having been informed by the research team. The participants' data will be fully protected and anonymized. The participants will have the right to withdraw from the study at any time without any consequence.

## Author Contributions

GM conceived and wrote the manuscript. PS and OW conceived the study and its design. ID, ERT, GM, IS, AT, and CV helped to conceive the study and its design. IS and AT helped to conduct parts of the background literature review. CV, PS, and OW helped to draft parts of the manuscript. All authors critically reviewed and approved the manuscript.

## Funding

This study was funded by the Swiss National Science Foundation (SNSF): Grant: No. 10001C_179292.

## Conflict of Interest

The authors declare that the research was conducted in the absence of any commercial or financial relationships that could be construed as a potential conflict of interest.

## Publisher's Note

All claims expressed in this article are solely those of the authors and do not necessarily represent those of their affiliated organizations, or those of the publisher, the editors and the reviewers. Any product that may be evaluated in this article, or claim that may be made by its manufacturer, is not guaranteed or endorsed by the publisher.
